# Supplementary material for: Metabolism-based targeting of MYC via MPC-SOD2 axis-mediated oxidation promotes cellular differentiation in group 3 medulloblastoma
Source: Nat Commun. 2023 May 2;14:2502. doi: 10.1038/s41467-023-38049-9 (PMC10154337; doi:10.1038/s41467-023-38049-9)
Supplement: Supplementary file 1 — Supplementary Information [file 41467_2023_38049_MOESM1_ESM.pdf]

Supplementary Information

Metabolism-based targeting of MYC via MPC-SOD2 axis-mediated oxidation promotes cellular differentiation in group 3 medulloblastoma

Martell et al.,

Supplementary Figure 1.

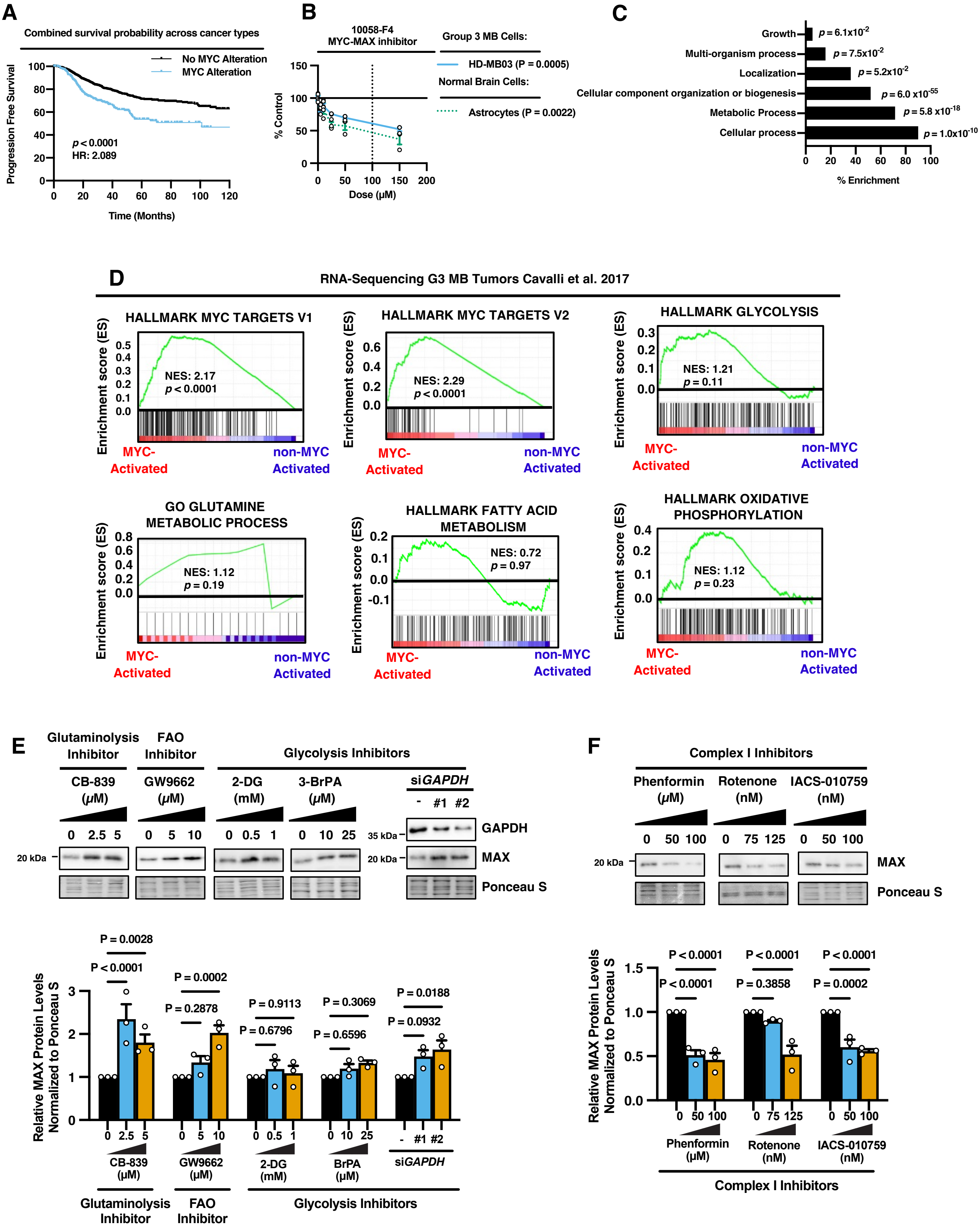

Supplementary Figure 1. (A) *Kaplan Meier* analysis of progression-free survival probability of patients with MYC-altered versus non-MYC altered tumors from Pan-cancer TCGA data, *N* = 10612 cases[1-3]. P-values and hazard ratio were determined using the logrank method. **(B)** HD-MB03 cells and normal human astrocytes were treated with 10058-F4 at various doses (5, 10, 25, 50 & 150 μM) and cells were counted after 24 hours. Values are plotted as mean of % of controls +/- SEM and p-values were calculated using two-sided student's t-test of maximum dose versus untreated controls. **(C)** Analysis of proteomics data of G3 MB tumors (*N* = 14) from Archer *et al*. 2018[4] and significantly differentially expressed proteins in MYC-activated versus non-MYC activated tumors (>2.0 fold-change and $p < 0.05$ based on two-sided student's t-test) were subjected to functional annotation clustering with gene ontology (GO) to identify differentially functionally enriched processes (p-values were determined using Fisher's exact t-test). **(D)** Analysis of RNA-sequencing data of G3 MB tumors (*N* = 144) from Cavalli *et al*. 2017[5] and gene set enrichment analysis (GSEA) was performed with Hallmark and gene ontology (GO) gene sets (enrichment scores and p-values are calculated using a weighted two-sided Kolmogorov–Smirnov-like statistic and normalized based on the size of the gene set to yield the normalized enrichment score, NES)[6,7]. **(E-F)** HD-MB03 cells were treated for 24 hours with indicated doses of metabolic inhibitors CB-839, GW9662, 2-DG, 3-BrPA, si*GAPDH* (two independent clones) and **(F)** complex-I inhibitors phenformin, rotenone, or IACS-010759, and MAX protein levels were monitored by immunoblotting. Graph represents mean values +/- SEM of densitometry quantification of blots from *N* = 3 experimental replicates. P-values are calculated using two-way ANOVA with Tukey's multiple comparisons. Source data provided in Source Data File.

Supplementary Figure 2.

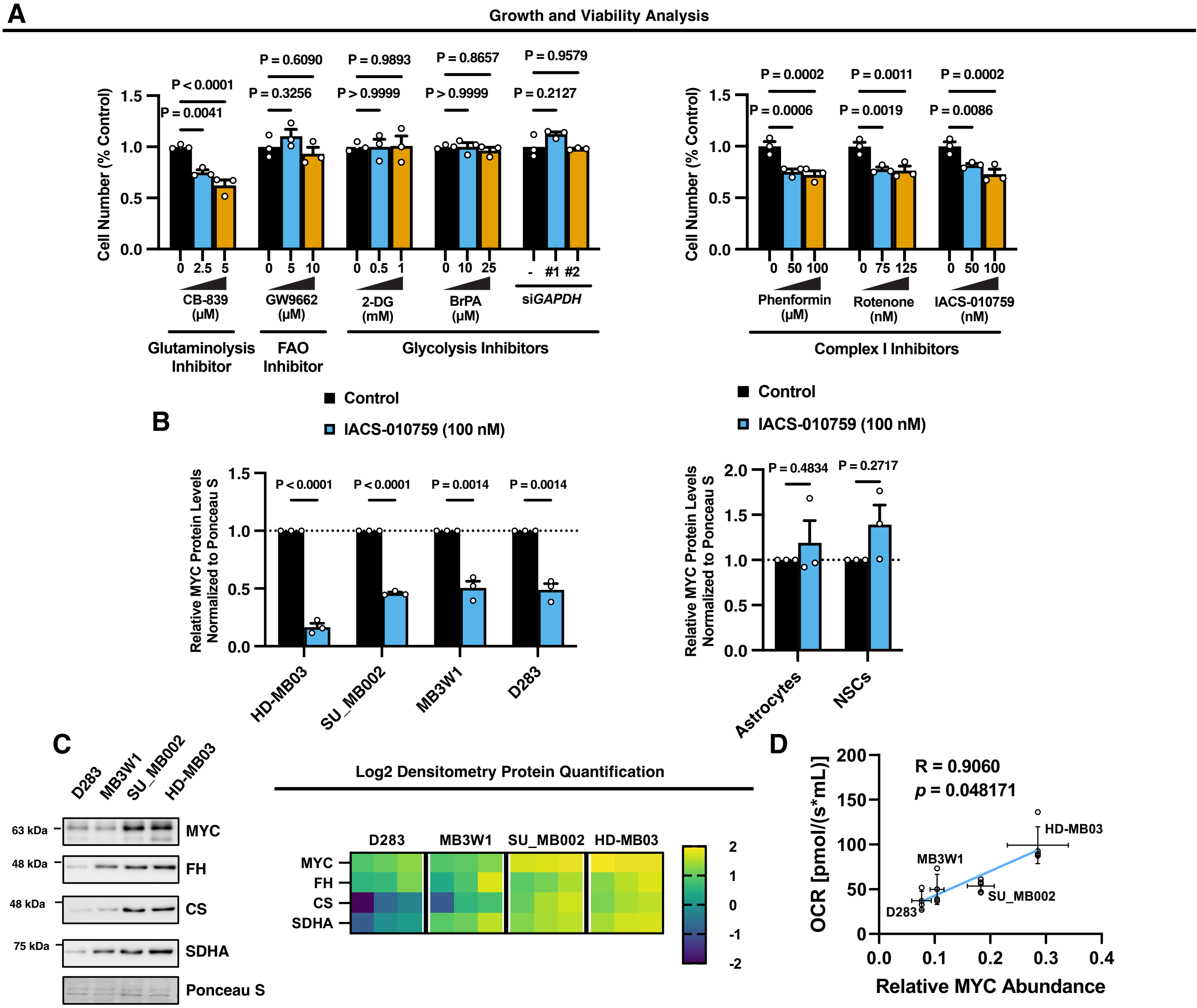

Supplementary Figure 2. (A) HD-MB03 G3 MB cells were treated with indicated doses of metabolic inhibitors CB-839, GW9662, 2-DG, 3-BrPA, si*GAPDH* (two independent clones), phenformin, rotenone, or IACS-010759 and counted after 24 hours. Values are plotted as mean percent cell number relative to respective vehicle controls +/- SEM from N = 3 experimental replicates. P-values were calculated using two-way ANOVA with Tukey's multiple comparisons. **(B)** Densitometry quantifications of western blots of MYC from N = 3 experimental replicates of HD-MB03, SU_MB002, MB3W1, D283, and normal human brain cells (astrocytes and neural stem cells; NSCs) that were treated with IACS-010759 for 24 hours. Values are plotted as mean +/- SEM and p-values are calculated using two-sided students t-test. **(C)** The protein levels of MYC and mitochondrial metabolic enzymes (fumarate hydratase, FH; citrate synthase, CS; and succinate dehydrogenase, SDHA) were compared in various G3 MB cells (D283, MB3W1, SU_MB002, and HD-MB03) by western blot analysis. Heat map displaying the log2 densitometry quantification of N = 3 experimental replicates. **(D)** The correlation between relative MYC protein abundance determined by western blot and the maximal oxygen consumption rate (OCR) of various G3 MB cells (D283, N = 4 experimental replicates; MB3W1, N = 4 experimental replicates; SU_MB002, N = 5 experimental replicates; and HD-MB03, N = 5 experimental replicates) was plotted as means +/- SEM of x and y values and Pearson correlation analysis was performed (R = 9.060, p = 0.048171). Source data provided in Source Data File.

Supplementary Figure 3.

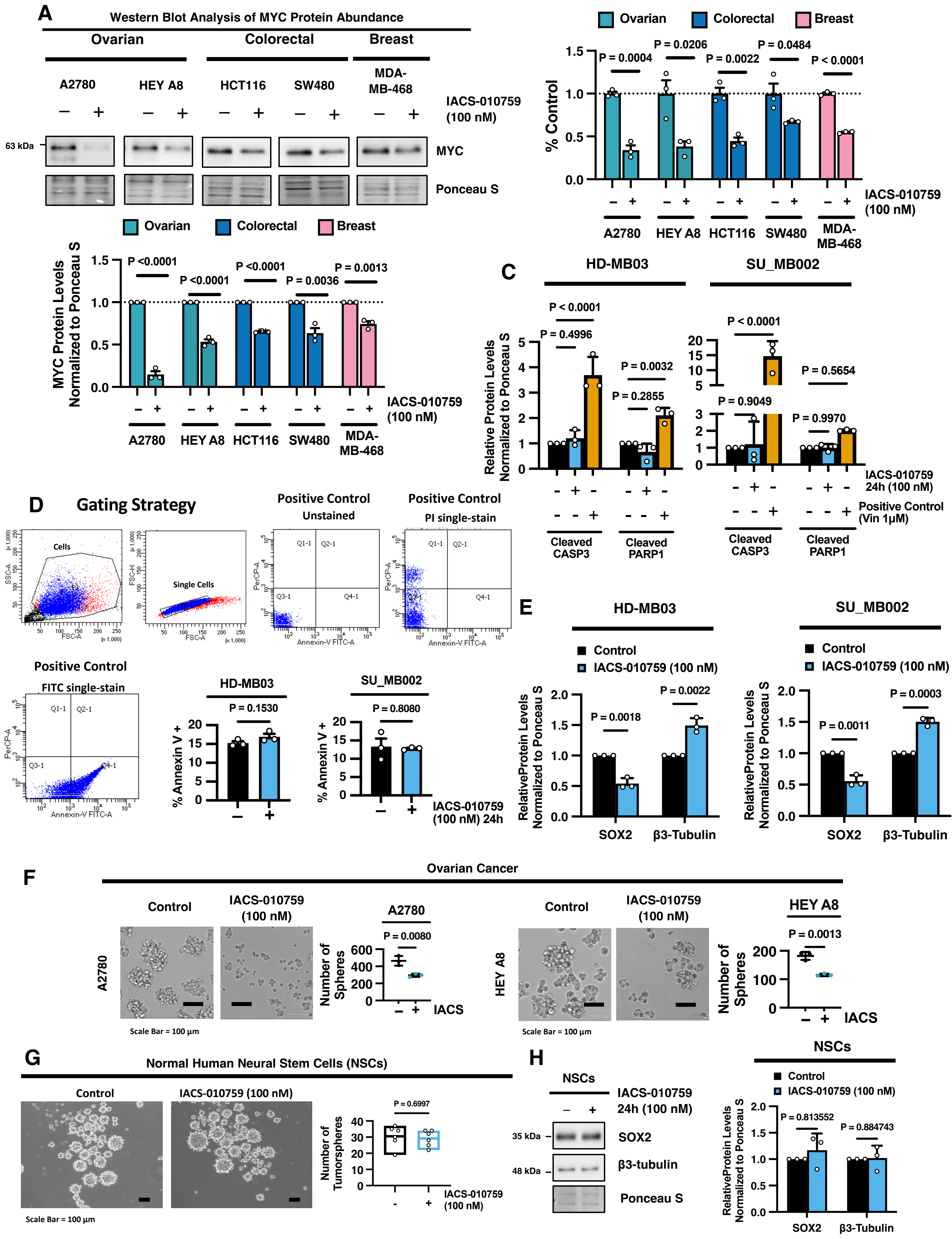

Supplementary Figure 3. (**A-B**) Various cancer cell lines from ovarian (A2780 and HEYA8), colorectal (HCT116 and SW40), and breast (MDA-MB-468) cancer were treated with 100 nM of IACS-010759 and **(A)** subjected to immunoblot analysis for MYC and graph represents mean densitometry quantification +/- SEM from N = 3 blots and **(B)** mean cell number +/- SEM counted after 24 hours (N = 3 experimental replicates). P-values were calculated using two-sided student's t-test relative to respective vehicle controls. **(C)** Densitometry quantification of western blots of cleaved caspase-3 and cleaved PARP1 presented as mean +/- SEM from N = 3 experimental replicates of HD-MB03 and SU_MB002 cells treated with IACS-010759 for 24 hours or positive control cytotoxic chemotherapy vincristine (1 μM). P-values were calculated using two-sided student's t-test relative to respective vehicle controls. N = 3 experimental replicates. **(D)** Gating strategy used for flow cytometry analysis in Figure 2F and percentage of Annexin V positive populations measured by flow cytometry presented as mean +/- SEM of vehicle control versus IACS-010759 treated (100 nM) HD-MB03 and SU_MB003 G3 MB cells. P-values were calculated using two-sided student's t-test relative to respective vehicle controls from N = 3 experimental replicates. **(E)** Densitometry quantification of western blots of SOX2 and β3-tubulin presented as mean +/- SEM from N = 3 experimental replicates of HD-MB03 and SU_MB002 cells treated with IACS-010759 for 24 hours. P-values were calculated using two-sided student's t-test relative to respective vehicle controls. **(F)** Representative tumorsphere images (Scale Bar = 100 μm) and quantification of total sphere number (>50 μm) from cancer cell lines (A2780 and HEYA8) treated with 100 nM of IACS-010759 presented as mean +/- SEM from N = 3 experimental replicates. P-values were calculated using two-sided student's t-test relative to respective vehicle controls. **(G)** Representative tumorsphere images (Scale Bar = 100 μm) and quantification of total sphere number (>50 μm) from neural stem cells (NSCs) treated with 100 nM of IACS-010759 presented as box-plot with the box limits at minima and maxima and centre line at mean from N = 6 experimental replicates. P-values were calculated using two-sided student's t-test relative to respective vehicle controls. **(H)** Western blots along with densitometry quantification presented as mean +/- SEM of SOX2 and β3-tubulin from N = 3 experimental replicates of normal human NSCs treated with IACS-010759 for 24 hours. P-values were calculated using two-sided student's t-test relative to respective vehicle controls. Source data provided in Source Data File.

Supplementary Figure 4.

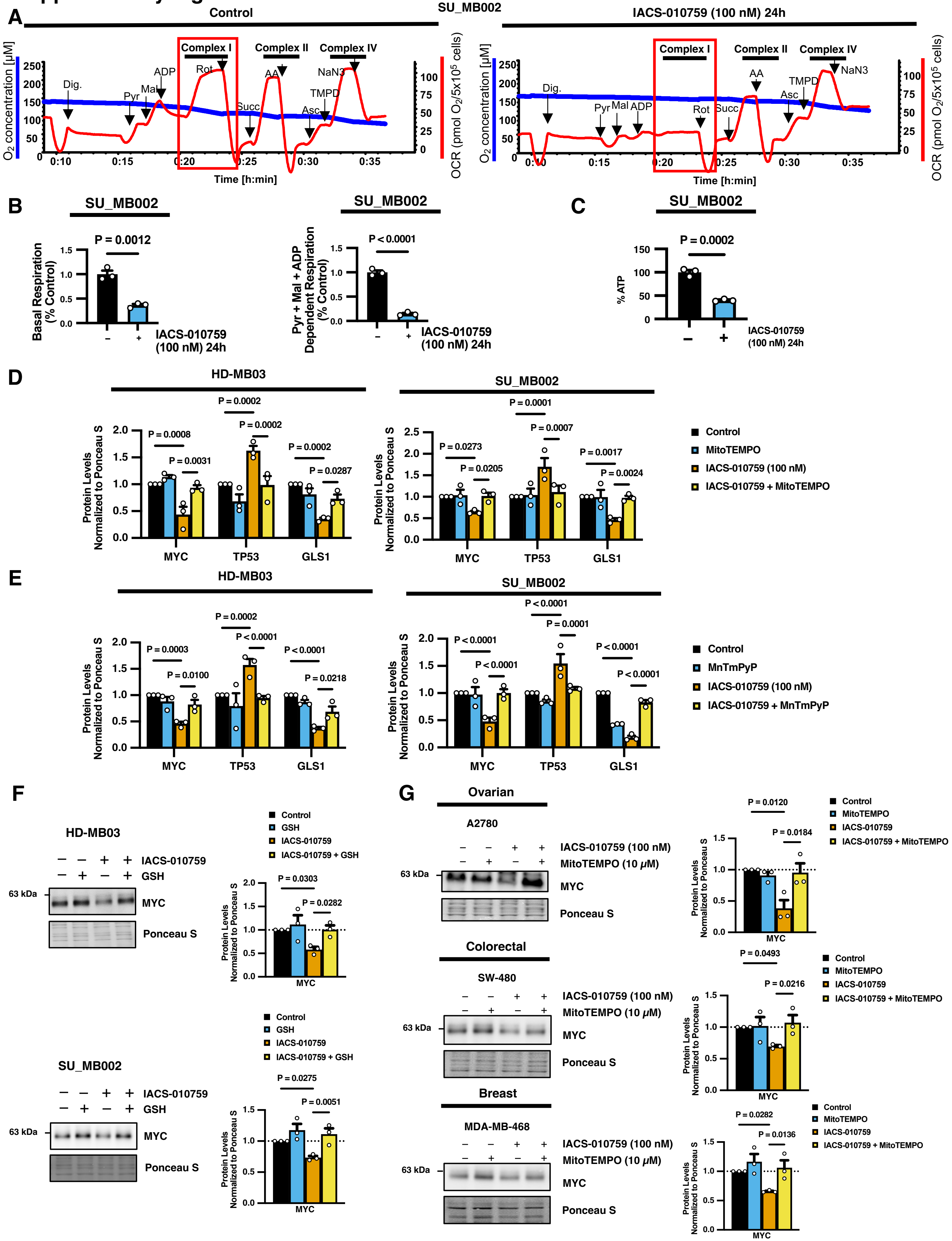

Supplementary Figure 4. (A-B) SU_MB002 cells were treated with IACS-010759 for 24 hours and subjected to Oroboros respirometry where **(A)** a representative tracing of oxygen consumption rate (OCR) substrate-specific oxygen consumption activity from *N* = 3 experimental replicates and **(B)** quantifications of basal and pyruvate-malate dependent OCR in SU_MB002 cells treated with IACS-010759 for 24 hours presented as mean +/- SEM where p-values were calculated using two-sided student's t-test relative to vehicle control. **(C)** ATP levels in SU_MB002 cells following IACS-010759 treatment for 24 hours presented as mean +/- SEM (*N* = 3 experimental replicates) where p-values were calculated using two-sided student's t-test relative to vehicle control. **(D-E)** Densitometry quantifications of western blots of MYC, TP53, and GLS1 presented as mean +/- SEM from *N* = 3 experimental replicates of HD-MB03 and SU_MB002 G3 MB cells treated with IACS-010759 along with either **(E)** MitoTEMPO or **(E)** MnTmPyP for 24 hours. P-values were calculated using two-way ANOVA with Tukey's multiple comparisons. **(F)** Representative western blots and densitometry quantifications of MYC presented as mean +/- SEM from *N* = 3 experimental replicates of HD-MB03 and SU_MB002 G3 MB cells treated with IACS-010759 along with the antioxidant glutathione (GSH). P-values were calculated using one-way ANOVA with Tukey's multiple comparisons. **(G)** Representative western blots and densitometry quantifications of MYC presented as mean +/- SEM from *N* = 3 experimental replicates of ovarian (A2780), colorectal (SW-480) and breast (MDA-MB-468) cells treated with IACS-010759 along with the antioxidant MitoTEMPO. P-values were calculated using one-way ANOVA with Tukey's multiple comparisons. Source data provided in Source Data File.

Supplementary Figure 5.

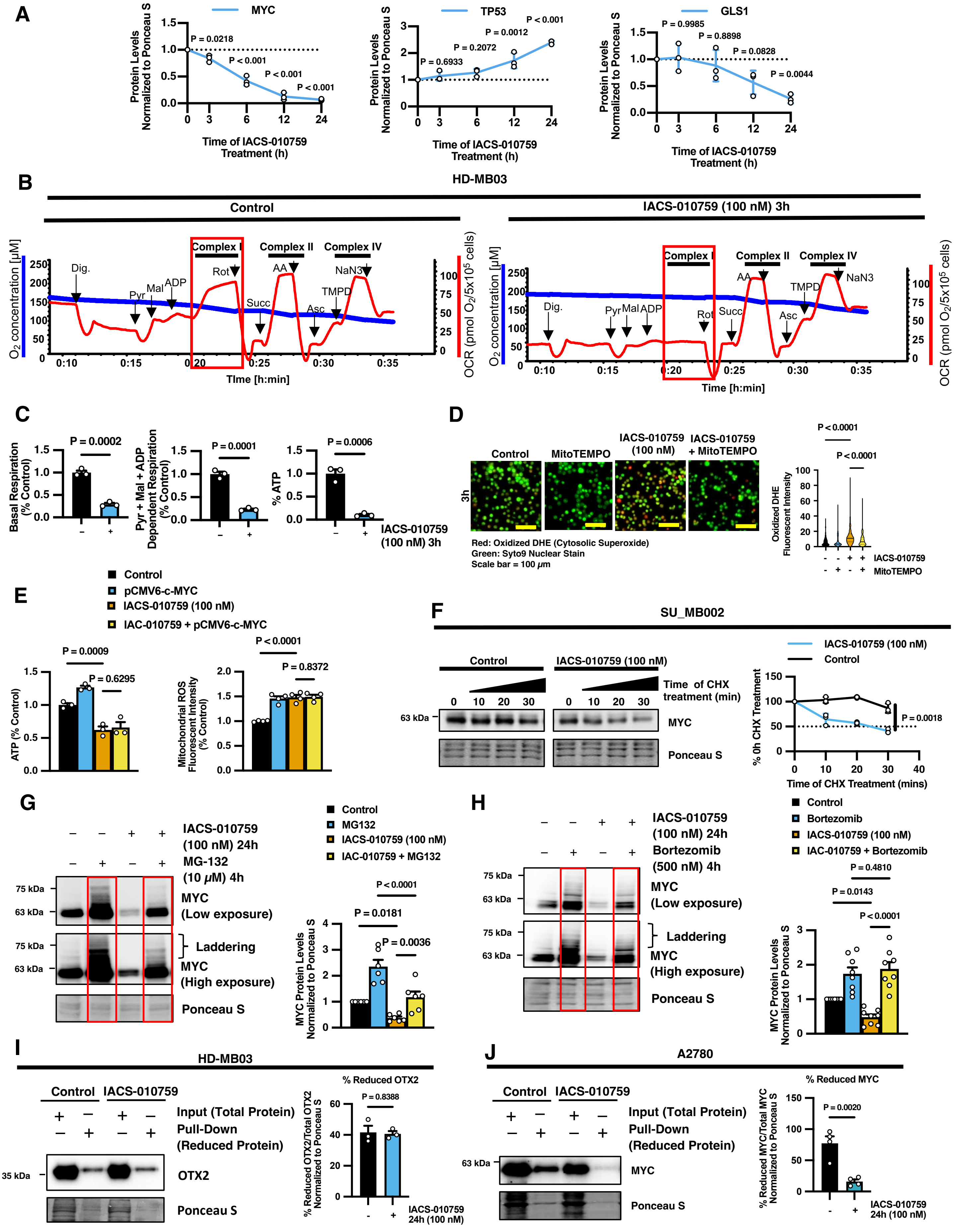

Supplementary Figure 5. (A) Densitometry quantification of western blots of MYC, TP53, and GLS1 in HD-MB03 cells treated with IACS-010759 at various time points plotted as mean +/- SEM. P-values were calculated using one-way ANOVA with Dunnett's multiple comparisons relative to respective vehicle controls. *N* = 3 experimental replicates. **(B-C)** HD-MB03 G3 MB cells were treated with IACS-010759 for 3 hours and subjected to Oroboros respirometry where **(B)** a representative tracing of OCR shows substrate-specific oxygen consumption and **(C)** baseline oxygen consumption, pyruvate-malate dependent oxygen consumption, and ATP levels were quantified and presented as mean +/- SEM from *N* = 3 experimental replicates. P-values were calculated using two-sided student's t-test relative to vehicle control. **(D)** Representative images (Scale Bar = 100 μm) of HD-MB03 cells treated with 100 nM of IACS-010759 and/or 10 μM of MitoTEMPO for 3 hours and labeled with DHE total superoxide stain and Syto9 green nuclear counter stain. Violin Plot represents the quantification of red fluorescent intensity per cell of *N* > 100 cells from *N* = 3 experimental replicates with solid line at mean and dashed lines at quartiles. P-values were calculated using two-way ANOVA with Tukey's multiple comparisons. **(E)** HD-MB03 cells were treated with IACS-010759 followed by exogenous overexpression of MYC for 48 hours and subjected to analysis of ATP levels (*N* = 3 experimental replicates) and mitochondrial superoxide production by MitoSOX staining (*N* = 4 experimental replicates) presented as mean +/- SEM. P-values were calculated using one-way ANOVA with Fisher's LSD test. **(F)** Representative western blots of MYC in SU_MB002 cells treated with IACS-010759 for 24 hours followed by the translation inhibitor cycloheximide (CHX) for 10, 20, and 30 minutes. Graphs represents densitometry quantification of blots from *N* = 3 experimental replicates presented as mean +/- SEM. P-values were calculated at final time point using two-sided student's t-test relative to respective vehicle control. **(G-H)** HD-MB03 cells were treated with 100 nM of IACS-010759 for 24 hours followed by the proteasome inhibitors **(G)** MG-132 (10 μM; *N* = 6 experimental replicates) or **(H)** Bortezomib (500 nM ; *N* = 8 experimental replicates) for 4 hours and subjected to immunoblot analysis for MYC. Graphs represents densitometry quantification of blots presented as mean +/- SEM. P-values were calculated using one-way ANOVA with Fisher's LSD test. **(I)** HD-MB03 cells treated with 100 nM of IACS-010759 and proteins with reduced cysteine thiols were labeled in lysates with maleimide-PEG2 biotin. Reduced proteins were precipitated with streptavidin agarose and subjected to immunoblot analysis for OTX2 relative to input lysates (no precipitation). Graphs represent densitometry quantification of blots from *N* = 3 experimental replicates presented as mean +/- SEM. P-values were calculated using two-sided student's t-test relative to vehicle control. **(J)** A2780 cells treated with 100 nM of IACS-010759 and proteins with reduced cysteine thiols were labeled in lysates with maleimide-PEG2 biotin. Reduced proteins were precipitated with streptavidin agarose and subjected to immunoblot analysis for MYC relative to input lysates (no precipitation). Graphs represent densitometry quantification of blots from *N* = 3 experimental replicates presented as mean +/- SEM. P-values were calculated using two-sided student's t-test relative to vehicle control. Source data provided in Source Data File.

Supplementary Figure 6.

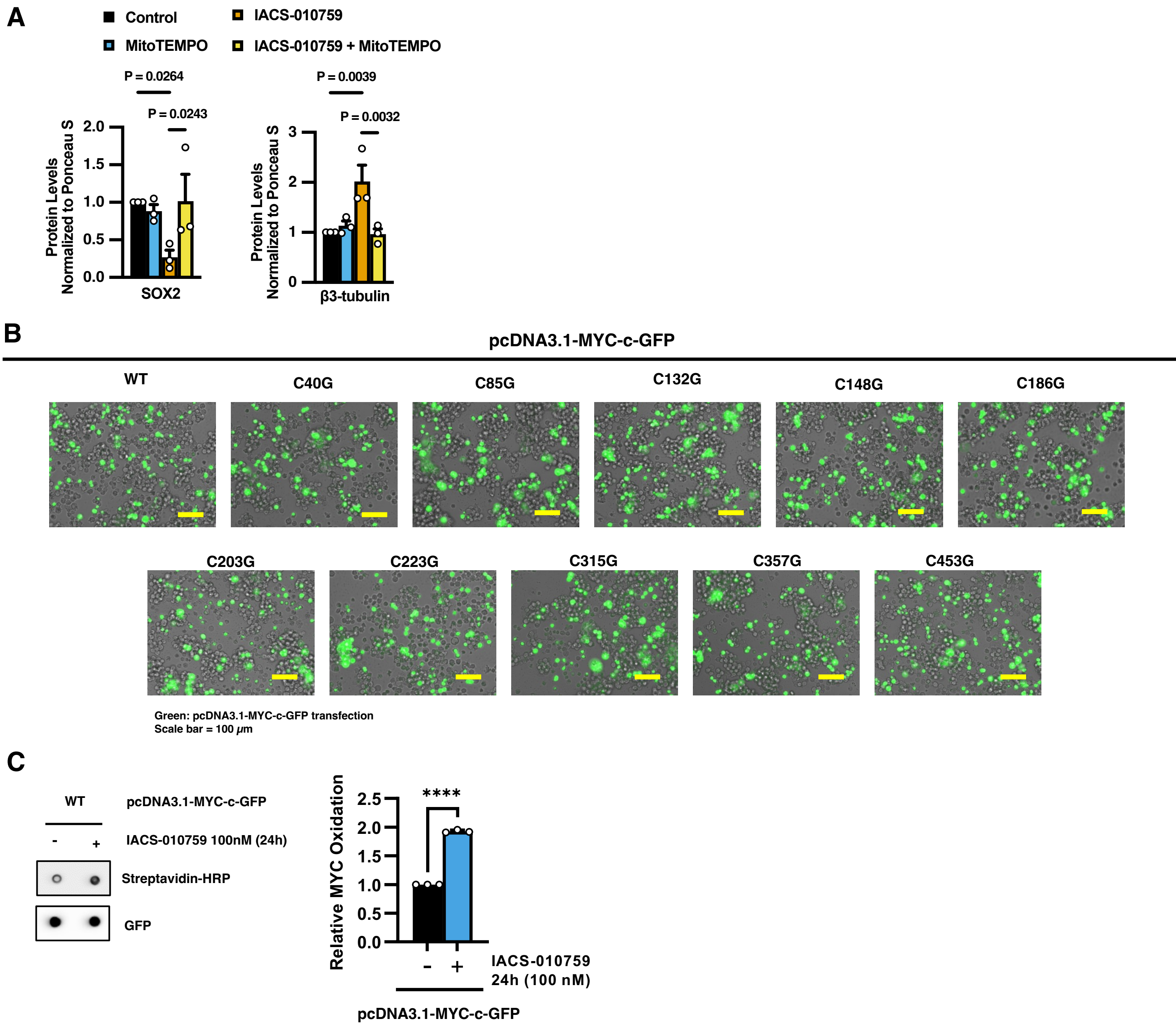

Supplementary Figure 6. (A) Densitometry quantification of western blots of SOX2 and β3-tubulin in HD-MB03 cells treated with IACS-010759 and/or MitoTEMPO from $N = 3$ experimental replicates presented as mean +/- SEM. P-values were calculated using one-way ANOVA with Fisher's LSD test. **(B)** Overlay of GFP fluorescent and brightfield microscopy of HD-MB03 cells expressing exogenous pcDNA3.1-MYC-c-eGFP MYC protein containing either wild-type MYC or point mutations in one of the 10 individual cysteine residues to monitor expression efficiency. Representative images (Scale Bar = 100 μm) from $N = 3$ experimental replicates. **(C)** Dot blot analysis of oxidized immunoprecipitated exogenous pcDNA3.1-MYC-c-eGFP containing wild-type MYC protein in control and IACS-010759 treated HD-MB03 cells. Densitometry quantification of $N = 3$ experimental replicates presented as mean +/- SEM. P-values were calculated using two-sided student's t-test relative to vehicle control. Source data provided in Source Data File.

Supplementary Figure 7.

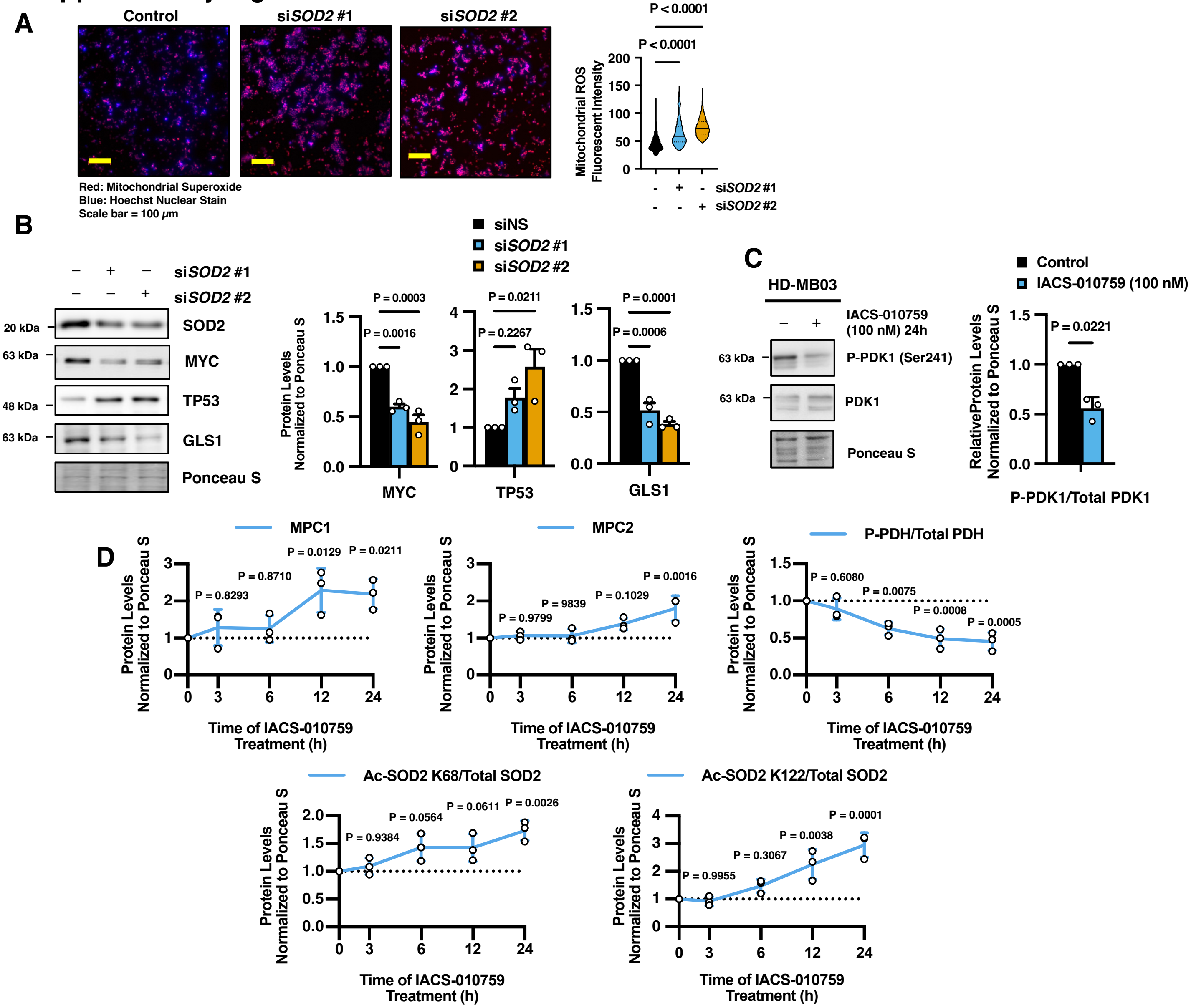

Supplementary Figure 7. (A) Representative images (Scale Bar = 100 µm) of MitoSOX red mitochondrial-specific superoxide stain with blue Hoechst nuclear counter stain in non-specific siRNA control HD-MB03 cells or si*SOD2* knockdown cells from two independent siRNA clones (#1 and #2). Violin plot represents quantification of fluorescent intensity per cell with solid line at mean and dashed lines at quartiles. P-values were calculated using one-way ANOVA with Tukey's multiple comparisons. N > 100 cells from N = 3 experimental replicates. **(B)** HD-MB03 cells with si*SOD2* knockdown from two independent siRNA clones (#1 and #2) were subjected to western blot analysis for SOD2, MYC, TP53 and GLS1. Densitometry quantification of western blots from N = 3 experimental replicates relative to non-specific siRNA control cells presented as mean +/- SEM. P-values were calculated using one-way ANOVA with Tukey's multiple comparisons. **(C)** HD-MB03 cells were treated with 100 nM of IACS-010759 and subjected to western blot analysis for P-PDK1 (Ser241) and total PDK1. Densitometry quantification of western blots from N = 3 experimental replicates presented as mean +/- SEM. P-values were calculated using two-sided student's t-test relative to vehicle control. **(D)** Densitometry quantification of western blots presented as mean +/- SEM from N = 3 experimental replicates of MPC1, MPC2, P-PDH (Ser293), PDH, Ac-SOD2 K68, Ac-SOD2 K122, and SOD2 from HD-MB03 cells treated with 100 nM IACS-010759 for indicated time points. P-values were calculated using one-way ANOVA with Dunnett's multiple comparisons relative to respective vehicle controls. Source data provided in Source Data File.

Supplementary Figure 8.

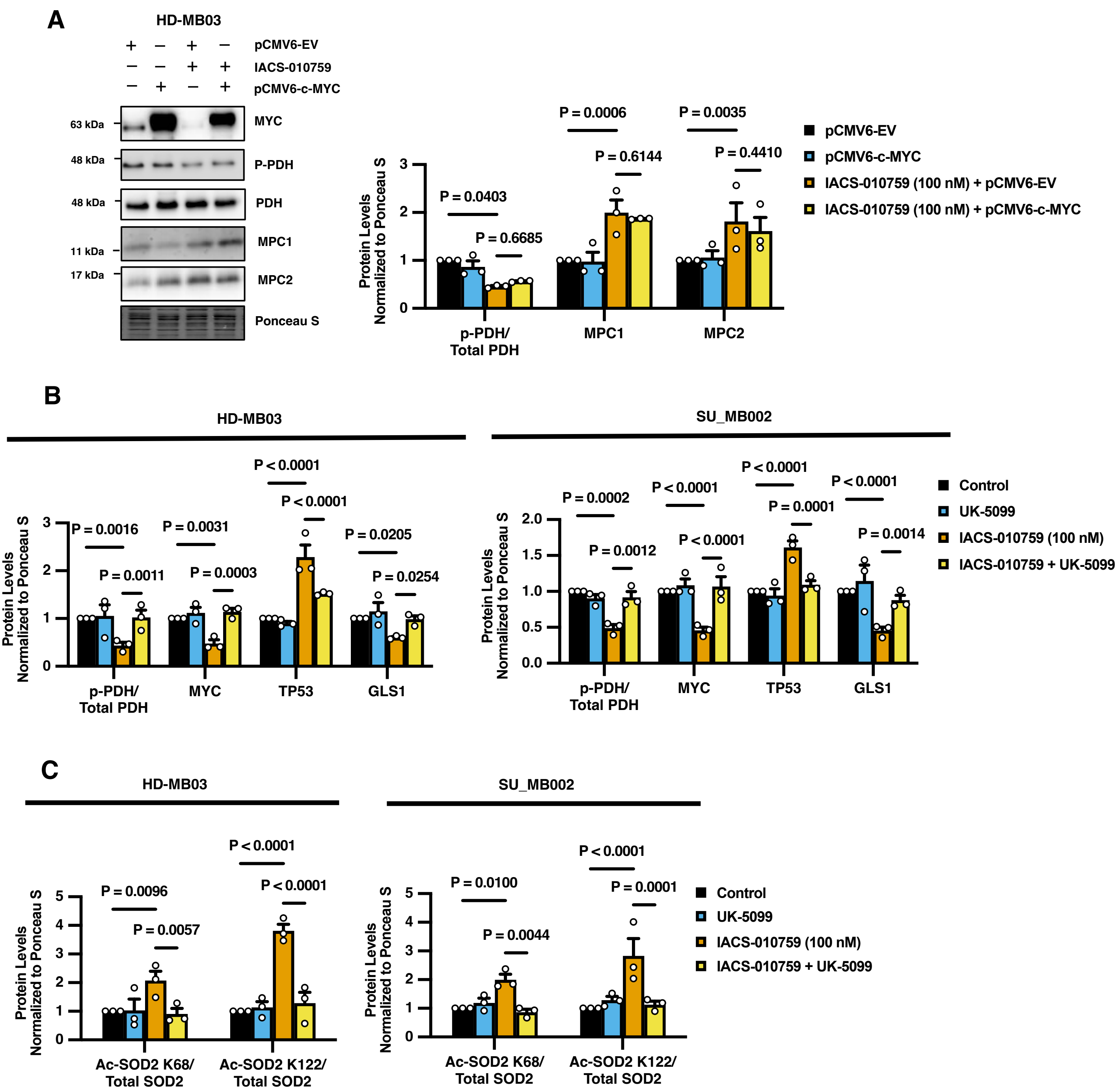

Supplementary Figure 8. (A) HD-MB03 cells were treated with 100 nM of IACS-010759 followed by exogenous overexpression of MYC for 48 hours and subjected to immunoblot analysis of P-PDH (Ser293), PDH, MPC1, and MPC2. Densitometry quantification presented as mean +/- SEM from *N* = 3 experimental replicates. P-values were calculated using two-way ANOVA with Fisher's LSD test. **(B-C)** Densitometry quantification of western blots presented as mean +/- SEM from *N* = 3 experimental replicates for **(B)** p-PDH (Ser293), PDH, MYC, TP53, GLS1, **(C)** Ac-SOD2 K68, Ac-SOD2 K122, and SOD2 in HD-MB03 and SU_MB002 G3 MB cells treated with IACS-010759 and/or UK-5099 for 24 hours. P-values were calculated using two-way ANOVA with Fisher's LSD test. Source data provided in Source Data File.

Supplementary Figure 9.

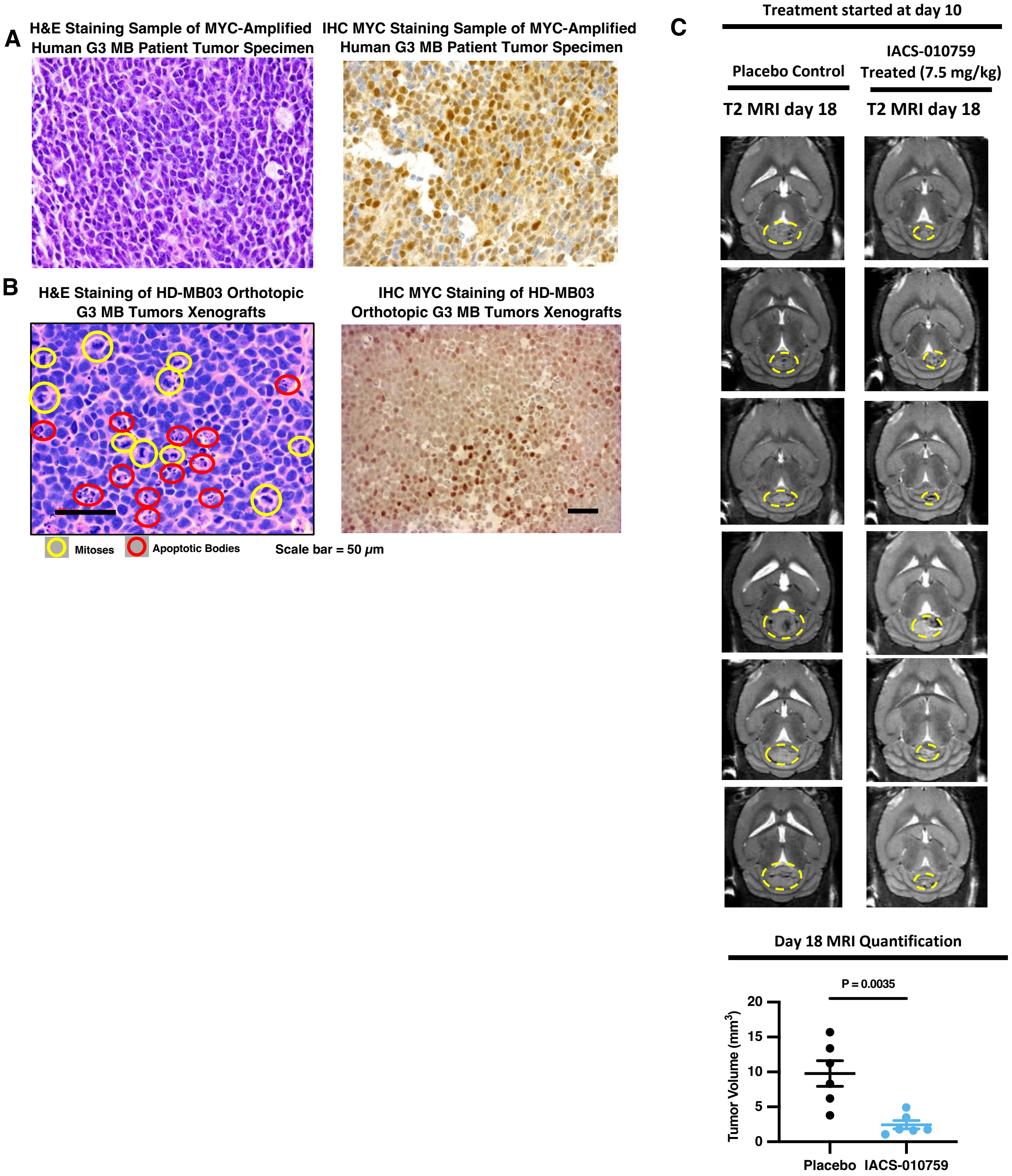

Supplementary Figure 9. (A) Representative images of anaplastic large cell G3 MB patient tumor specimen with H&E staining and immunohistochemistry (IHC) staining of MYC available open access from the r2genomics server in the Pediatric PDX (Olson) portal (Med-411FH)[8]. **(B)** Representative images of HD-MB03 orthotopic intracerebellar tumor xenografts with H&E staining (Scale Bar = 200 μm) and immunohistochemistry (IHC) staining of MYC (Scale Bar = 50 μm) from N = 5 control animals. **(C)** T2 MRI images of tumor masses from matching planes at 18-days post-surgery and H&E stained brain slices from mice that received either placebo control (0.5% methylcellulose; N = 6 animals) or 7.5 mg/kg Q2Dx5 IACS-010759 treatment (N = 6 animals) starting at 10-days post-surgery. Quantification of tumor volume from 18-day MRI images presented as mean +/- SEM. P-values were calculated using two-sided student's t-test relative to vehicle control. Source data provided in Source Data File.

Supplementary Table 1. List of treatment compounds used in this study

	Vendor	Catalog No.	Dose(s)
**10058-F4**	Abcam	ab145065	5, 10, 25, 50, & 150 µM
**CB-839**	Selleckchem	S7655	2.5 & 5 µM
**GW-9662**	Sigma-Aldrich	M6191	5 & 10 µM
**2-DG**	Sigma-Aldrich	D8375	0.25, 0.5, 1, 2, 3 mM
**3-BrPA**	Sigma-Aldrich	16490	10, 25, 50, 100, & 250 µM
**Phenformin**	Selleckchem	S2542	10, 50, 100, 250, & 500 µM
**Rotenone**	Sigma-Aldrich	R8875	10, 50, 100, 500, & 1000 nM
**IACS-010759 (*in vitro*)**	Selleckchem	S8731	100 nM (unless otherwise indicated)
**IACS-010759 (*in vivo)***	MedChem Express	HY-112037	7.5 mg/kg
**si*GAPDH* clone #1**	Invitrogen; silencer select	4390824; assay ID: s5572	50 nM
**si*GAPDH* clone #2**	Invitrogen; silencer select	4390824; assay ID: s5573	50 nM
**Negative control siRNA**	Invitrogen	AM4635	50 nM
**si*SOD2* clone #1**	Invitrogen; silencer select	4390824; assay ID: n323027	50 nM
**si*SOD2* clone #2**	Invitrogen; silencer select	4390824; assay ID: s13268	50 nM
**Vincristine**	Selleckchem	S1241	1 µM
**pCMV6-EV**	Origene	RC201611	1.5 µg/mL
**pCMV6-c-MYC**	Origene	PS100001	1.5 µg/mL
**pcDNA3.1(+)-MYC-c-eGFP**	Genscript	SC1844	1.5 µg/mL
**pcDNA3.1(+)-MYC-C40G-c-eGFP**	Genscript	SC1844	1.5 µg/mL
**pcDNA3.1(+)-MYC-C85G-c-eGFP**	Genscript	SC1844	1.5 µg/mL
**pcDNA3.1(+)-MYC-C132G-c-eGFP**	Genscript	SC1844	1.5 µg/mL
**pcDNA3.1(+)-MYC-C148G-c-eGFP**	Genscript	SC1844	1.5 µg/mL
**pcDNA3.1(+)-MYC-C186G-c-eGFP**	Genscript	SC1844	1.5 µg/mL
**pcDNA3.1(+)-MYC-C203G-c-eGFP**	Genscript	SC1844	1.5 µg/mL
**pcDNA3.1(+)-MYC-C223G-c-eGFP**	Genscript	SC1844	1.5 µg/mL
**pcDNA3.1(+)-MYC-C315G-c-eGFP**	Genscript	SC1844	1.5 µg/mL
**pcDNA3.1(+)-MYC-C357G-c-eGFP**	Genscript	SC1844	1.5 µg/mL
**pcDNA3.1(+)-MYC-C453G-c-eGFP**	Genscript	SC1844	1.5 µg/mL
**MitoTEMPO**	Cayman	16621	10 µM
**MnTmPyp**	Adipogen	AG-CR1-0026	50 µM
**Glutathione**	Santa Cruz	sc-203974	100 µM
**Cyclohexamide**	Sigma-Aldrich	C7698	1.25 µM
**MG-132**	Sigma-Aldrich	M8699	10 µM
**Bortezomib**	Selleckchem	S1013	500 nM
**UK-5099**	Cayman	16980	10 µM

Supplementary Table 2. List of antibodies used for immunoblotting

	Vendor	Catalog No.	Dilution
**CPTA1**	Cell Signaling Technology	97361S	1:1000
**PPAR$_{\gamma}$**	Cell Signaling Technology	2435S	1:1000
**c-MYC**	Cell Signaling Technology	18583S	1:1000
**MAX**	Cell Signaling Technology	4739S	1:1000
**GAPDH**	DSHB	DSHB-hGAPDH-2G7	1:250
**Cleaved Caspase-3**	Cell Signaling Technology	9661S	1:1000
**PARP1**	Santa Cruz	sc-8007	1:1000
**SOX2**	Cell Signaling Technology	3579S	1:1000
**β3-tubulin**	R&D Systems	MAB1195	1:1000
**P53**	Santa Cruz	sc-126	1:1000
**GLS1**	Cell Signaling Technology	88964S	1:1000
**Poly Ubiquitin**	Cell Signaling Technology	3936S	1:1000
**OTX2**	Abcam	ab21990	1:1000
**GFP**	Santa Cruz	sc-9996	1:1000
**SOD2**	Cell Signaling Technology	13141S	1:1000
**Acetylated SOD2 K68**	Abcam	ab137037	1:1000
**Acetylated SOD2 K122**	Abcam	ab214675	1:1000
**MPC1**	Cell Signaling Technology	14462	1:1000
**MPC2**	Cell Signaling Technology	46141	1:1000
**P-PDH**	Cell Signaling Technology	31866S	1:1000
**PDH**	Cell Signaling Technology	3205S	1:1000
**P-PDK1**	Cell Signaling Technology	3438S	1:1000
**PDK1**	Cell Signaling Technology	3062S	1:1000
**Goat anti-mouse HRP**	Jackson Immunoresearch	115-035-003	1:10,000
**Goat anti-rabbit HRP**	Jackson Immunoresearch	111-035-003	1:10,000

Supplementary Table 3. List of antibodies used for immunohistochemistry

	Vendor	Catalog No.	Dilution
**c-MYC**	Abcam	ab32072	1:100
**Ki67**	Cell Signaling Technology	9449S	1:800
**SOX2**	Abcam	ab97959	1:100
**β3-tubulin**	R&D Systems	MAB1195	1:250
**P53**	Santa Cruz	sc-126	1:50
**GLS1**	Cell Signaling Technology	56750	1:200
**8-Hydroxy-2'-deoxyguanosine**	Abcam	ab48508	1:50
**4-Hydroxynonenal**	Abcam	ab48506	1:25
**MPC2**	ThermoFisher	PA5-63246	1:20
**MPC1**	ThermoFisher	PA5-60929	1:500
**Acetylated SOD2 K68**	Abcam	ab137037	1:100
**Sheep anti-mouse Biotin**	Jackson Immunoresearch	515-065-003	1:500
**Sheep anti-rabbit Biotin**	Jackson Immunoresearch	111-065-144	1:500

Supplementary Table 4. List of primer sequences used in this study

	Forward Primer	Reverse Primer
MYC	CCTGGTGCTCCATGAGGAGAC	CAGACTCTGACCTTTTGCCAGG
SOX2	GGGAAATGGAGGGGTGCAAAAGAGG	TTGCGTGAGTGTGGATGGGATTGGTG
NANOG	TGAACCTCAGCTACAAACAG	TGGTGGTAGGAAGAGTAAAG
NES	TCAAGATGTCCCTCAGCCTGGA	AAGCTGAGGGAAGTCTTGGAGC
TUBB3	GGCCTCTTCTCACAAGTACG	CCACTCTGACCAAAGATGA
MAP2	AGGCTGTAGCAGTCCTGAAAGG	CTTCCTCCACTGTGACAGTCTG
NEUROD1	GGTGCCTTGCTATTCTAAGACGC	GCAAAGCGTCTGAACGAAGGAG
NEUROG1	GCCTCCGAAGACTTCACCTACC	GGAAAGTAACAGTGTCTACAAAGG
MILIP	AGAACCGCGAAAGGCTACTG	CACTTAAAGCCGGTCGTGGA
AIMP2	ACCACCAATGCGCTGGACTTGA	AGGACCCTGAAGTGCTCACAGA
BYSL	CTCTCCAACTGGGAGCAAATCC	TGCGTTCCTTCAGGTTAGAGGC
CBX3	GCTGACAAACCAAGAGGATTTGC	CAGCACCAAGTCTGCCTCATCT
CDK4	CCATCAGCACAGTTCGTGAGGT	TCAGTTCGGGATGTGGCACAGA
DCTPP1	TCCATCAGCCTCGGAATCTCCT	CCTCTTGAAGGGCTGCCCGTT
DDX18	GATGTGGCAGCGAGAGGACTAG	GGCGCAAAATGAGCAAGGCATG
DUSP2	TGTGGAGGACAACCAGATGGTG	GAGGTATGCCAGACAGATGGTG
EXOSC5	GAACGGAAGCTGCTGATGTCCA	GGTAGAAACGGAAGACGTGTTGC
FARSA	TGGCTGAGTTCCACCAGATCGA	TTGTAGGCTGGCTTGAAGCGGA
GNL3	TTTCCCAGGCTGATGCTCGACA	GCAGTTTGGCAGCACCTTCAAC
GRWD1	CCATCTTCTCCTTCGCTGGACA	AGGTGTCCAGAGGTGGATGTTC
HK2	GAGTTTGACCTGGATGTGGTTGC	CCTCCATGTAGCAGGCATTGCT
HSPD1	TGCCAATGCTCACCGTAAGCCT	AGCCTTGACTGCCACAACCTGA
HSPE1	GCTGAAACTGTAACCAAAGGAGG	TCTCCAACTTTCACGCTAACTGG
IMP4	CACCTCATCACACACGGCTTCT	GTAGTCGTCCTGGTTTGCGAAG
IPO4	CCTCGCAAGTTGTACGCAATGC	TGTCCAAGAGGCACCGACTTCA
LAS1L	AAGGCAGCGAAGAGGTGGATTC	GCACCGTAAACTGCTCCTCTTC
MAP3K6	CGCCACAAGAACATAGTGCGCT	ACTGATGGTGCTCTCGTTGTCC
MCM4	CTTGCTTCAGCCTTGGCTCCAA	GTCGCCACACAGCAAGATGTTG
MCM5	GACTTACTCGCCGAGGAGACAT	TGCTGCCTTTCCCAGACGTGTA
MPHOSPH10	GGAAGTGACAGCACAGAAGAGG	CCAGTTGAAGGGTGGTTTCCTC
MRTO4	ACAGCAAGCTGAAGGACATCCG	GACCTGGTGCAGGTTGTCTTTG
MYBBP1A	CAGTTCGCAGACCTCCTGTTGA	TCCAGCTCCTTCAGAGTCTGCA
NDUFAF4	CAGCAAGATGAAGCCCTCTGTC	CACATCTTTTAGAAACGACAGCAG
NIP7	AGAGCAGTCCTTCCTGTATGGG	GCAGTCTTGTGTAGATTTGGCTG
NOC4L	GGAACAATGCCTTCACGCTGCT	TCAGGTGAGCAACCTTCCAGGT
NOLC1	GTAGCAGTGATGACTCAGAGGAG	CTGGAGGAATCCTCACTGCTAG
NOP16	CCTATGTGCTGAATGACCTGGAG	CGTGGTTCTCTACCATGTAGCG
NOP2	CTGTCAATGCGACCTCCAAGAC	GAAAGCGGGTAAAACCTTCCTGG
NOP56	GGCTAAGGCTATTCTGGATGCC	TGTGTAGGCTCTGGCGGTATTC
NPM1	GCCAGTGCATATTAGTGGACAGC	GGAACCTTGCTACCACCTCCAG
PA2G4	GCTCACCTTTGTGCTGAAGCTG	GCTGCTTCAACTGGTGTGACAG

PES1	TCGTGTGGATCACTCCCTATGC	GGTTGAGCAACTGGTAAAGGCG
PHB	AAGCGGTGGAAGCCAAACAGGT	GCCAGTGAGTTGGCAATCAGCT
PLK1	GCACAGTGTCAATGCCTCCAAG	GCCGTACTTGTCCGAATAGTCC
PLK4	GACACCTCAGACTGAAACCGTAC	GTCCTTCTGCAAATCTGGATGGC
PPAN	AGCAGAAACGGCTTGCCAAGTC	GAACCCACTGAGTTTGTCGTCG
PPRC1	ATTGAGGCATCGGACCTGTCCA	CCTGAGTTTCCTACAGCCAAGC
PRMT3	CACTGTCTGCTGAAGCCGCATT	GTAGATGACGAGCAGGTTCTGAC
PUS1	TCAACAGCCACCTTCCCTCTCA	GCAATAGGTCCTGGCATCACATC
RABEPK	CACATCATCGGCAGCCATTGGA	GGAGGATTTCCAAGTGTCTCTGG
RCL1	CCTGTGAGGAAGGTCTTGAAGC	GTTCGCCATCTGAGGTGACACA
RRP12	AGTGCCTCCTACACATCGTGAG	GAGCAGTGCAAAAGCGTTCTTCC
RRP9	GTTGGTGGCAAAAGAGATCCAGG	CTTTGGCAGCAGAGAAGATGGC
SLC19A1	CTTTGCCACCATCGTCAAGACC	GGACAGGATCAGGAAGTACACG
SLC29A2	GGATCTTGACCTGGAGAAGGAG	GTGAAGACCAACACAAGGCACAG
SORD	GCCGATACAATCTGTCACCTTCC	CGCCTTCCTCAAAGGTGACATTG
SRM	CTACCAGGACATCCTCGTCTTC	AGAGGCAGGTTGGCGATCATCT
SUPV3L1	ACTTCTCAGCAAAGTCTGGAGTG	TGGCTGAACTGTCACACGCTCT
TBRG4	CAGCCTGACTTCTGGTGAGGTG	GTGATGTCCTGCGCTCTGTTCA
TCOF1	CAGGAAGACTCTGAGAGCAGTG	CCCTTTTGCTGAAGGTGCTCTG
TFB2M	GGGAAAACCAAGTAGACCTCCAC	TTTCGAGCGCAACCACTTTGGC
TMEM97	GAGCAAGATGGTGTCAGGAACC	AGGTTGCAGTGAGCCGAGATCA
UNG	CCACACCAAGTCTTCACCTGGA	CCGTGAGCTTGATTAGGTCCATG
UTP20	GAGACTTTCCAGACCATCACCTC	ACTCATCAGGCACATGCTGGCA
WDR43	CCTCCACAAACCGAGCAAGTAG	GCTATTCGTCTGGAGGTCTTCC
WDR74	GAAAACAGGCGGCGAACTTCAC	TGCTGAAGTGCTTCACCGTCCT
GLS1	CAGAAGGCACAGACATGGTTGG	GGCAGAAACCACCATTAGCCAG

SUPPLEMENTARY REFERENCES

1 Hoadley, K. A. et al. Cell-of-Origin Patterns Dominate the Molecular Classification of 10,000 Tumors from 33 Types of Cancer. Cell 173, 291-304 e296, doi:10.1016/j.cell.2018.03.022 (2018).

2 Cerami, E. et al. The cBio cancer genomics portal: an open platform for exploring multidimensional cancer genomics data. Cancer Discov 2, 401-404, doi:10.1158/2159-8290.CD-12-0095 (2012).

3 Gao, J. et al. Integrative analysis of complex cancer genomics and clinical profiles using the cBioPortal. Sci Signal 6, pl1, doi:10.1126/scisignal.2004088 (2013).

4 Archer, T. C. et al. Proteomics, Post-translational Modifications, and Integrative Analyses Reveal Molecular Heterogeneity within Medulloblastoma Subgroups. Cancer Cell 34, 396-410 e398, doi:10.1016/j.ccell.2018.08.004 (2018).

5 Cavalli, F. M. G. et al. Intertumoral Heterogeneity within Medulloblastoma Subgroups. Cancer Cell 31, 737-754 e736, doi:10.1016/j.ccell.2017.05.005 (2017).

6 Subramanian, A. et al. Gene set enrichment analysis: a knowledge-based approach for interpreting genome-wide expression profiles. Proc Natl Acad Sci U S A 102, 15545-15550, doi:10.1073/pnas.0506580102 (2005).

7 Liberzon, A. et al. The Molecular Signatures Database (MSigDB) hallmark gene set collection. Cell Syst 1, 417-425, doi:10.1016/j.cels.2015.12.004 (2015).

8 Brabetz, S. et al. A biobank of patient-derived pediatric brain tumor models. Nat Med 24, 1752-1761, doi:10.1038/s41591-018-0207-3 (2018).
