# Supplementary material for: Metabolism-based targeting of MYC via MPC-SOD2 axis-mediated oxidation promotes cellular differentiation in group 3 medulloblastoma
Source: Nat Commun. 2023 May 2;14:2502. doi: 10.1038/s41467-023-38049-9 (PMC10154337; doi:10.1038/s41467-023-38049-9)
Supplement: Supplementary file 3 — Reporting Summary [file 41467_2023_38049_MOESM3_ESM.pdf]

nature portfolio

Corresponding author(s): Tanveer Sharif

Last updated by author(s): Apr 6, 2023

Reporting Summary

Nature Portfolio wishes to improve the reproducibility of the work that we publish. This form provides structure for consistency and transparency in reporting. For further information on Nature Portfolio policies, see our Editorial Policies and the Editorial Policy Checklist.

Statistics

For all statistical analyses, confirm that the following items are present in the figure legend, table legend, main text, or Methods section.

n/a	Confirmed	
[ ]	[x]	The exact sample size (*n*) for each experimental group/condition, given as a discrete number and unit of measurement
[ ]	[x]	A statement on whether measurements were taken from distinct samples or whether the same sample was measured repeatedly
[ ]	[x]	The statistical test(s) used AND whether they are one- or two-sided<br>*Only common tests should be described solely by name; describe more complex techniques in the Methods section.*
[x]	[ ]	A description of all covariates tested
[ ]	[x]	A description of any assumptions or corrections, such as tests of normality and adjustment for multiple comparisons
[ ]	[x]	A full description of the statistical parameters including central tendency (e.g. means) or other basic estimates (e.g. regression coefficient) AND variation (e.g. standard deviation) or associated estimates of uncertainty (e.g. confidence intervals)
[ ]	[x]	For null hypothesis testing, the test statistic (e.g. *F*, *t*, *r*) with confidence intervals, effect sizes, degrees of freedom and *P* value noted<br>*Give P values as exact values whenever suitable.*
[x]	[ ]	For Bayesian analysis, information on the choice of priors and Markov chain Monte Carlo settings
[x]	[ ]	For hierarchical and complex designs, identification of the appropriate level for tests and full reporting of outcomes
[ ]	[x]	Estimates of effect sizes (e.g. Cohen's *d*, Pearson's *r*), indicating how they were calculated

Our web collection on statistics for biologists contains articles on many of the points above.

Software and code

Policy information about availability of computer code

Data collection	No software was used
Data analysis	Prism 9.0, GSEA (Broad Institute, v4.0), DAVID 2021 (https://david.ncifcrf.gov/summary.jsp), Oroboros DatLab software (v6.0), ImageJ (v1.8.0_172, NIH), FlowJo™ v10 Software, BioRender (https://www.biorender.com/)

For manuscripts utilizing custom algorithms or software that are central to the research but not yet described in published literature, software must be made available to editors and reviewers. We strongly encourage code deposition in a community repository (e.g. GitHub). See the Nature Portfolio guidelines for submitting code & software for further information.

Data

Policy information about availability of data

All manuscripts must include a data availability statement. This statement should provide the following information, where applicable:

- Accession codes, unique identifiers, or web links for publicly available datasets
- A description of any restrictions on data availability
- For clinical datasets or third party data, please ensure that the statement adheres to our policy

All data generated or analysed during this study are included in this manuscript (and its supplementary information files). Analysis of TCGA pan-cancer studies was performed using the cBioPortal online bioinformatics platform for cancer genomics. The TCGA pan-cancer atlas (a combination of studies from 32 cancers with a total of 10967 samples) was queried for alterations of MYC and the alteration frequency was reported and summarized based on cancer type and downloaded from BioPortal for Cancer Genomics (https://www.cbioportal.org/. Patient MB tumor RNA-sequencing data was accessed from Cavalli et al. 20175 using the R2 genomics

and visualization online platform (http://r2.amc.nl), accessible from GEO (accession number GSE85217).Patient MB tumor proteomics data was accessed from Archer et al. 2018 (doi: 10.1016/j.ccell.2018.08.004). Gene sets used in GSEA analysis were accessed from the Molecular Signatures Database (mSigDB, Broad Institute) https://www.gsea-msigdb.org/gsea/msigdb/.

Human research participants

Policy information about studies involving human research participants and Sex and Gender in Research.

Reporting on sex and gender	N/A
Population characteristics	N/A
Recruitment	N/A
Ethics oversight	N/A

Note that full information on the approval of the study protocol must also be provided in the manuscript.

Field-specific reporting

Please select the one below that is the best fit for your research. If you are not sure, read the appropriate sections before making your selection.

- [x] Life sciences
- [ ] Behavioural & social sciences
- [ ] Ecological, evolutionary & environmental sciences

For a reference copy of the document with all sections, see nature.com/documents/nr-reporting-summary-flat.pdf

Life sciences study design

All studies must disclose on these points even when the disclosure is negative.

Sample size	No power calculations were utilized to determine required sample size for in vitro and in vivo experiments. For in vivo studies, sample size was determined based on availability of animals and cost of the drug. For in vitro studies, all experiments were performed on a minimum of 3 independent biological replicates for each cell line with the majority of cell culture-based assays also being repeated with an additional 1-2 independent cell lines accordingly. Figure legends indicate sample sizes for each experiment. Data from all experiments were analyzed and p-values from statistical tests used to assess statistical significant and appropriateness of sample sizes. For patient sample transcriptome and proteome analysis sample size was based on the number of samples already present in publicly available datasets.
Data exclusions	No data are excluded.
Replication	For vitro/molecular experiments (ie. tumorsphere assays, qPCR, immunoblotting, microscopy, etc.), studies were carried out in 3 or greater independent biological replicates for each cell line with the majority also being replicated in 1-2 additional cell lines for reproducibility. All statistics were performed on independent biological replicates. All attempts at replication were successful and provide an overall reflection of each individual experiment's findings. Two independent vivo studies were performed on 10 and 12 male NOD-SCID gamma mice equally divided to randomly receive either placebo control or experimental IACS-010759 treatment. Tumors from all animals were collected and IHC analysis was performed on slides from all individual animals. IHC for all samples were processed in parallel with representative images displayed and quantification of all samples present in the main figures.
Randomization	Following intracerebellar transplantation, NOD SCID gamma mice were randomly distributed into groups for IACS-010759 treatment. For all other experiments, cells/samples were randomly assigned into groups
Blinding	Blinding was performed during tissue preparation for IHC. Remaining studies were not blinded because the individual(s) performing the experiment/analysis were also responsible for preparing the samples. Instead, the experiments were replicated as described above and/or repeated in 1-2 additional cell lines.

Reporting for specific materials, systems and methods

We require information from authors about some types of materials, experimental systems and methods used in many studies. Here, indicate whether each material, system or method listed is relevant to your study. If you are not sure if a list item applies to your research, read the appropriate section before selecting a response.

Materials & experimental systems

n/a	Involved in the study
☐	☒ Antibodies
☐	☒ Eukaryotic cell lines
☒	☐ Palaeontology and archaeology
☐	☒ Animals and other organisms
☒	☐ Clinical data
☒	☐ Dual use research of concern

Methods

n/a	Involved in the study
☒	☐ ChIP-seq
☐	☒ Flow cytometry
☐	☒ MRI-based neuroimaging

Antibodies

Antibodies used

For immunoblotting:
GFP (B-2) Santa Cruz sc-9996 (1:1000)
CPTA1 Cell Signaling Technology 97361S (1:1000)
PPARγ Cell Signaling Technology 2435S (1:1000)
c-MYC Cell Signaling Technology 18583S (1:1000)
MAX Cell Signaling Technology 4739S (1:1000)
GAPDH DSHB DSHB-hGAPDH-2G7 (1:250)
Cleaved Caspase-3 Cell Signaling Technology 9661S (1:1000)
PARP1 Santa Cruz sc-8007 (1:1000)
SOX2 Cell Signaling Technology 3579S (1:1000)
β3-tubulin Clone # TuJ-1 R&D Systems MAB1195 (1:1000)
P53 Santa Cruz sc-126 (1:1000)
GLS1 Cell Signaling Technology 88964S (1:1000)
Poly Ubiquitin Cell Signaling Technology 3936S (1:1000)
OTX2 Abcam ab21990 (1:1000)
SOD2 Cell Signaling Technology 13141S (1:1000)
Acetylated SOD2 K68 Abcam ab137037 (1:1000)
Acetylated SOD2 K122 Abcam ab214675 (1:1000)
MPC1 Cell Signaling Technology 14462 (1:1000)
MPC2 Cell Signaling Technology 46141 (1:1000)
P-PDH Cell Signaling Technology 31866S (1:1000)
PDH Cell Signaling Technology 3205S (1:1000)
P-PDK1 Cell Signaling Technology 3438S (1:1000)
PDK1 Cell Signaling Technology 3062S (1:1000)
Goat anti-mouse HRP Jackson Immunoresearch 115-035-003 (1:10,000)
Goat anti-rabbit HRP Jackson Immunoresearch 111-035-003 (1:10,000)

For immunohistochemistry:
c-MYC Abcam ab32072 (1:100)
Ki67 Cell Signaling Technology 9449S (1:800)
SOX2 Abcam ab97959 (1:100)
β3-tubulin # TuJ-1 R&D Systems MAB1195 (1:250)
P53 Santa Cruz sc-126 (1:50)
GLS1 Cell Signaling Technology 56750 (1:200)
8-Hydroxy-2'-deoxyguanosine Abcam ab48508 (1:50)
4-Hydroxynonenal Abcam ab48506 (1:25)
MPC2 ThermoFisher PA5-63246 (1:20)
MPC1 ThermoFisher PA5-60929 (1:500)
Acetylated SOD2 K68 Abcam ab137037 (1:100)
Sheep anti-mouse Biotin Jackson Immunoresearch 515-065-003 (1:500)
Sheep anti-rabbit Biotin Jackson Immunoresearch 111-065-144 (1:500)

Validation

Antibodies were validated for the specified assays by the manufactors.

From the vendors:

CPTA1 Cell Signaling Technology 97361S (Western blot): Monoclonal antibody is produced by immunizing animals with a synthetic peptide corresponding to residues surrounding Leu696 of human CPT1A protein. CPT1A (E3Y1V) Rabbit mAb recognizes endogenous levels of total CPT1A protein. This antibody does not cross-react with CPT1B, CPT1C, or CPT2.Western blot analysis of extracts from various cell lines using CPT1A (E3Y1V) Rabbit mAb shows the absence of detected CPT1A expression in SK-MEL-5 cell extracts is consistent with RNAseq expression profiling data, confirming specificity of the antibody for CPT1A.

PPARγ Cell Signaling Technology 2435S (Western blot): PPARγ (C26H12) Rabbit mAb is produced by immunizing rabbits with a synthetic peptide corresponding to residues surrounding Asp69 of human PPARγ. PPARγ (C26H12) Rabbit mAb detects endogenous

levels of total PPARγ protein. Western blot analysis of extracts from NIH/3T3 and 3T3-L1 cells (differentiated 6 days into adipocytes) detects PPARγ.

c-MYC Cell Signaling Technology 18583S (Western blot): Monoclonal antibody is produced by immunizing animals with recombinant protein specific to the amino terminus of human c-Myc protein. c-Myc (E5Q6W) Rabbit mAb recognizes endogenous levels of total c-Myc protein. Western blot analysis of extracts from various cell lines (SCLC-21H, Raji, KG-1a, IMR-32, A20, BaF3, RBL-2H3) detects c-Myc.

MAX Cell Signaling Technology 4739S (Western blot): Polyclonal antibodies are produced by immunizing animals with a synthetic peptide corresponding to residues near the amino terminus of Max. Antibodies are purified by protein A and peptide affinity chromatography. Max (S20) Antibody detects endogenous levels of total Max protein (Species Reactivity: Human, Mouse, Rat). Western blot analysis of extracts from various cell lines detects Max in Raji, C6, and Pys-2.

Cleaved Caspase-3 Cell Signaling Technology 9661S (Western blot): Polyclonal antibodies are produced by immunizing animals with a synthetic peptide corresponding to amino-terminal residues adjacent to (Asp175) in human caspase-3. Cleaved Caspase-3 (Asp175) Antibody detects endogenous levels of the large fragment (17/19 kDa) of activated caspase-3 resulting from cleavage adjacent to Asp175. This antibody does not recognize full length caspase-3 or other cleaved caspases. This antibody detects non-specific caspase substrates by western blot. Non-specific labeling may be observed by immunofluorescence in specific sub-types of healthy cells in fixed-frozen tissues (e.g. pancreatic alpha-cells). Nuclear background may be observed in rat and monkey samples. Western blot analysis of extracts from HeLa, NIH/3T3 and C6 cells untreated, staurosporine-treated (3hrs, 1 µM in vivo) or cytochrome c-treated (1hr, 0.25 mg/ml in vitro) detects cleaved caspase-3.

SOX2 Cell Signaling Technology 3579S (Western blot): Monoclonal antibody is produced by immunizing animals with a synthetic peptide corresponding to amino acids surrounding Gly179 of human Sox2. Sox2 (D6D9) XP® Rabbit mAb detects endogenous levels of Sox2 protein. Western blot analysis of extracts from NTERA2 and NCCIT cells detects Sox2.

GLS1 Cell Signaling Technology 88964S (Western blot): Polyclonal antibodies are produced by immunizing animals with a synthetic peptide corresponding to residues surrounding Gly116 of human glutaminase-1/GLS1 protein. Antibodies are purified by protein A and peptide affinity chromatography. Glutaminase-1/GLS1 Antibody recognizes endogenous levels of total glutaminase-1/GLS1 protein. Western blot analysis of extract from human kidney detects GLS1.

Poly Ubiquitin Cell Signaling Technology 3936S (Western blot): Monoclonal antibody is produced by immunizing animals with 1-76 full length bovine ubiquitin. Ubiquitin (P4D1) Mouse mAb detects ubiquitin, polyubiquitin and ubiquitinated proteins. This antibody may cross-react with recombinant NEDD8. Western blot analysis of 293 and HeLa cells, untreated or treated with the 26S proteasome inhibitor MG132 (50 µM, 90 minutes) detects poly ubiquitin.

SOD2 Cell Signaling Technology 13141S (Western blot): Monoclonal antibody is produced by immunizing animals with a synthetic peptide corresponding to residues near the amino terminus of human SOD2 protein. SOD2 (D3X8F) XP® Rabbit mAb recognizes endogenous levels of total SOD2 protein. Western blot analysis of extracts from various cell lines (HeLa, 293, NIH/3T3, mIMCD-3, C2C12, Raw 264.7, NBT-II, INS-1, Hep G2, COS-7) detects SOD2.

MPC1 Cell Signaling Technology 14462 (Western blot): Monoclonal antibody is produced by immunizing animals with a synthetic peptide corresponding to residues near the carboxy terminus of human MPC1 protein. MPC1 (D2L9I) Rabbit mAb recognizes endogenous levels of total MPC1 protein. This antibody does not cross-react with MPC2 protein. Western blot analysis of extracts from various cell lines (Hep2G, Huh7, COLO 205, MDA-MB-435, K-562) detects MPC1.

MPC2 Cell Signaling Technology 46141 (Western blot): Monoclonal antibody is produced by immunizing animals with a synthetic peptide corresponding to residues surrounding Asn33 of human MPC2 protein. MPC2 (D4I7G) Rabbit mAb recognizes endogenous levels of total MPC2 protein. This antibody does not cross-react with MPC1 protein. Western blot analysis of extracts from various cell lines (Huh7, COLO 205, MDA-MB-435, K-562, RPMI 8226, Raw 264.7, B-TC-6) detects MPC2.

P-PDH Cell Signaling Technology 31866S (Western blot): Polyclonal antibody is produced by immunizing animals with a synthetic phosphopeptide corresponding to residues surrounding Ser293 of human pyruvate dehydrogenase α1 protein. Phospho-Pyruvate Dehydrogenase α1 (Ser293) Antibody recognizes endogenous levels of pyruvate dehydrogenase α1 protein only when phosphorylated at Ser293 residue. Based on amino acid sequence comparisons, this antibody is predicted to detect endogenous levels of pyruvate dehydrogenase α2 protein only when phosphorylated at Ser291 residue. Western blot analysis of extracts from HeLa cells, untreated (-) or treated (+) with calf intestinal alkaline phosphatase (CIP)/λ phosphatase, and BaF3 cells, detected Phospho-Pyruvate Dehydrogenase α1 (Ser293).

PDH Cell Signaling Technology 3205S (Western blot): Monoclonal antibody is produced by immunizing animals with a synthetic peptide corresponding to the sequence of human pyruvate dehydrogenase. Pyruvate Dehydrogenase (C54G1) Rabbit mAb detects endogenous levels of total pyruvate dehydrogenase α1 subunit. Western blot analysis of extracts from various cell lines (HepG2, 293, HeLa, A204) detects Pyruvate Dehydrogenase.

P-PDK1 Cell Signaling Technology 3438S (Western blot): Monoclonal antibody is produced by immunizing animals with a synthetic phosphopeptide corresponding to residues around Ser241 of human PDK1. Phospho-PDK1 (Ser241) (C49H2) Rabbit mAb detects PDK1 only when phosphorylated at Ser241. Western blot analysis of extracts from PC3 cells, HCT116 wild-type and HCT116 PDK1 -/- cells using Phospho-PDK1.

PDK1 Cell Signaling Technology 3062S (Western blot): Polyclonal antibodies are produced by immunizing animals with a synthetic peptide corresponding to residues surrounding the carboxy terminus of human PDK1. Antibodies are purified by protein A and peptide affinity chromatography. PDK1 Antibody detects endogenous levels of total PDK1 protein. Western blot analysis of extracts from SW-13, NIH/3T3, Jurkat and PC12 cells detects PDK1.

Ki67 Cell Signaling Technology 9449S (Immunohistochemistry): Monoclonal antibody is produced by immunizing animals with a synthetic peptide corresponding to residues near the amino terminus of human Ki-67 protein. Ki-67 (8D5) Mouse mAb recognizes endogenous levels of total Ki-67 protein. Immunohistochemical analysis of paraffin-embedded human breast carcinoma, human colon carcinoma, and human ovarian serous adenocarcinoma detects Ki-67.

GLS1 Cell Signaling Technology 56750 (Immunohistochemistry): Monoclonal antibody is produced by immunizing animals with a synthetic peptide corresponding to residues surrounding Gly116 of human glutaminase-1/GLS1 protein. Glutaminase-1/GLS1 (E9H6H) XP® Rabbit mAb recognizes endogenous levels of total glutaminase-1/GLS1 protein. This antibody does not cross-react with glutaminase-2/GLS2 protein. Immunohistochemical analysis of paraffin-embedded human appendix and human ductal breast carcinoma detects Glutaminase-1/GLS1. Immunohistochemical analysis of paraffin-embedded human colon carcinoma using Glutaminase-1/GLS1 (E9H6H) XP® Rabbit mAb compared to concentration-matched Rabbit (DA1E) mAb IgG XP® Isotype Control.

OTX2 Abcam ab21990 (Western blot): ab21990 is raised against the C-terminal region of the Human Otx2 protein. Reacts with: Mouse, Chicken, Cow, Human, Xenopus laevis. This product is batch tested in Y79 cell lysate, recombinant Otx1 and overexpressed Otx2. All batches should detect endogenous Otx2, overexpressed Otx2 and recombinant or overexpressed Otx1.

Acetylated SOD2 K68 Abcam ab137037 (Western blot & Immunohistochemistry): ab137037 only detects SOD2/MnSOD when acetylated at Lysine 68. According to BLAST analysis, the antibody might cross-react with Fer (Uniprot P70451) isoform 3 in mouse samples. No experiment has been done to confirm this possibility. Reacts with: Mouse, Rat, Human. Immunohistochemistry (Formalin/PFA-fixed paraffin-embedded sections) analysis of human clear cell carcinoma kidney tissue labelling SOD2 (acetyl K68) with purified ab137037 at 1/150. Heat mediated antigen retrieval was performed using Tris/EDTA buffer pH 9. ab97051, a goat anti-rabbit IgG H&L (HRP) was used as the secondary antibody (1/500). Negative control using PBS instead of primary antibody. Counterstained with hematoxylin. Western blot of Anti-SOD2/MnSOD (acetyl K68) antibody [EPVANR2] (ab137037) at 1/10000 dilution (purified) in Trichostatin A treated SOD2 transfected HEK-293T lysates with acetyl peptide, Trichostatin A treated SOD2 transfected HEK-293T lysates with non-acetyl peptide, Trichostatin A treated SOD2 transfected HEK-293T lysates.

Acetylated SOD2 K122 Abcam ab214675 (Western blot): Rabbit monoclonal [NCI-R156-33] to SOD2/MnSOD (acetyl K122). Reacts with: Recombinant fragment. Predicted to work with: Human.Flag-SOD2/MnSOD (acetyl K122) was immunoprecipitated from HEK293T cells, transfected with Flag-tagged SOD2/MnSOD (acetyl K122) expression vector and treated with 1µM Trichostatin A (TSA) for 48 h, with anti-Flag antibody. Western blot was performed from the immunoprecipitate using ab214675 at 1/200 dilution. SIRT3 with NAD+ decreased SOD2/MnSOD acetylation in vitro. The expression profile observed is consistent with what has been described in the literature (PMID: 21172655; PMID: 25852572).

c-MYC Abcam ab32072 (Immunohistochemistry): This antibody is specific for endogenous c-Myc. It does not detect Myc tag. Reacts with: Mouse, Rat, Human. Immunohistochemistry (Formalin/PFA-fixed paraffin-embedded sections) analysis of human diffuse large B cell lymphoma tissue labelling c-Myc with purified ab32072 at 1/500. Heat mediated antigen retrieval was performed using Tris/EDTA buffer pH 9. Goat Anti-Rabbit IgG H&L (HRP) (ab97051) secondary antibody was used as the secondary antibody (1/500). Negative control using PBS instead of primary antibody. Counterstained with hematoxylin.

SOX2 Abcam ab97959 (Immunohistochemistry): Rabbit polyclonal to SOX2. Reacts with: Mouse, Rat, Human. IHC image of SOX2 staining in Human brain glioma formalin fixed paraffin embedded tissue section, performed on a Leica Bond™ system using the standard protocol F. The section was pre-treated using heat mediated antigen retrieval with sodium citrate buffer (pH6, epitope retrieval solution 1) for 20 mins. The section was then incubated with ab97959, 1µg/ml, for 15 mins at room temperature and detected using an HRP conjugated compact polymer system. DAB was used as the chromogen. The section was then counterstained with haematoxylin and mounted with DPX.

Anti-PDHA1 (phospho S293) Abcam ab92696 (Immunohistochemistry): Rabbit polyclonal to PDHA1 (phospho S293). Reacts with: Human. This antibody gave a positive result in IHC in the following FFPE tissue: Human lung adenocarcinoma. ICC/IF: HepG2 cells.

8-Hydroxy-2'-deoxyguanosine Abcam ab48508 (Immunohistochemistry): Mouse monoclonal [N45.1] to 8-Hydroxy-2'-deoxyguanosine. Nineteen analogues of 8-Hydroxy-2'-deoxyguanosine {guanosine (G), 7-methyl-G, 6-SH-G, 8-bromo-G, dA, dC, dT, dI, dU, dG, O6-methyl-dG, 8-OHdA, guanine (Gua), O6-methyl-Gua, 8-OHGua, uric acid, Urea, creatine, creatinine} demonstrate no cross-reactivity. Only 8-sulfhydryl-G and 8-OHG demonstrate minimal cross-reactivity (less than 1%). Reacts with: Species independent. ab48508 staining 8-Hydroxy-2'-deoxyguanosine in human colon tissue sections by Immunohistochemistry (IHC-P - paraformaldehyde-fixed, paraffin-embedded sections). Tissue was fixed with paraformaldehyde and antigen retrieval was by heat mediation in a TE buffer. Samples were incubated with primary antibody (1/50) for 30 mins at 20°C. A HRP-conjugated Goat anti-mouse IgG was used as the secondary antibody.

4-Hydroxynonenal Abcam ab48506 (Immunohistochemistry): Mouse monoclonal [HNEJ-2] to 4 Hydroxynonenal. Reacts with: Species independent. ab48506 at a 1/25 dilution staining 4-Hydroxy-2-Nonenal in mouse heart tissue sections by Immunohistochemistry (paraffin embedded) incubated for 15 hours at +4°C. Fixed with formaldehyde, heat mediated antigen retrieval step performed using citrate buffer. Blocked using 5% serum for 20 minutes at 20°C. Secondary used undiluted polyclonal Goat anti-mouse IgG conjugated to Alexa Fluor 594.

PARP1 Santa Cruz sc-8007 (Western blot): raised against amino acids 764-1014 mapping at the C-terminus of PARP of human origin. PARP1 Antibody (F-2) is recommended for detection of full-length PARP-1 and the C-terminal cleavage product of PARP-1 of human origin by WB, IP, IF, IHC(P) and ELISA. Western blot analysis of PARP-1 expression in Jurkat, NTERA-2 cl.D1, and SJRH30 whole cell lysates.

P53 Santa Cruz sc-126 (Western blot & Immunohistochemistry): N-terminal epitope mapping between amino acid residues 11-25 of p53 of human origin. p53 Antibody (DO-1) is recommended for detection of wild type and mutant p53 under denaturing and non-denaturing conditions of mouse, rat and human origin by WB, IP, IF, IHC(P) and FCM. Western blot analysis of p53 expression in

SW480, A549, and HUV-EC-C whole cell lysates. Immunoperoxidase staining of formalin fixed, paraffin-embedded human pancreas adenocarcinoma tissue showing nuclear staining of tumor cells.

MPC2 ThermoFisher PA5-63246 (Immunohistochemistry): Recombinant protein corresponding to Human BRP44 (MPC2). Species Reactivity: Human. Immunohistochemical staining of BRP44 in human stomach using a BRP44 Polyclonal Antibody (Product # PA5-63246) shows moderate to strong cytoplasmic positivity in glandular cells.

MPC1 ThermoFisher PA5-60929 (Immunohistochemistry): Recombinant protein corresponding to Human BRP44L (MPC1). This Antibody was verified by Relative expression to ensure that the antibody binds to the antigen stated. Detection of differential expression levels of BRP44L demonstrates antibody specificity. Immunohistochemical analysis of BRP44L using anti-BRP44L Polyclonal Antibody (Product # PA5-60929), shows significant staining of BRP44L in human heart muscle and shows minimal or weak staining in human pancreas tissues. The relative expression levels of BRP44L within each tissue is shown using RNA-Seq.

GAPDH DSHB DSHB-hGAPDH-2G7 (Western blot): Positive Tested Species Reactivity: Human. Recommended Applications: Western Blot. Obtained from the Developmental Studies Hybridoma Bank, created by the NICHD of the NIH and maintained at The University of Iowa, Department of Biology, Iowa City, IA 52242.

Neuron-specific β3-tubulin Clone # TuJ-1 R&D Systems MAB1195 (Western blot & Immunohistochemistry): Immunogen: Rat brain-derived microtubules. Detects mammalian and chicken neuron-specific beta -III tubulin but not other beta -tubulin isotypes in Western blots. Western blot shows lysates of human brain (cerebellum) tissue, human brain (hypothalamus) tissue, mouse brain (cerebellum) tissue, and mouse brain (stem) tissue. beta -III Tubulin was detected in immersion fixed paraffin-embedded sections of human brain (cerebellum) using Mouse Anti-Neuron-specific beta -III Tubulin Monoclonal Antibody.

Eukaryotic cell lines

Policy information about cell lines and Sex and Gender in Research

Cell line source(s)

Human MB cells were obtained from collaborators as kind gifts. Dr. Till Milde provided HD-MB03, a treatment-naïve large cell/anaplastic G3 MB cell model isolated from a 3-year old male patient during surgical intervention. SU_MB002 cells were provided by Dr. Yoon-Jae Cho and were derived from an autopsy specimen of the leptomeningeal compartment from a child with treatment-refractory, metastatic G3 MB after receiving only cyclophosphamide treatment. MB3W1 anaplastic G3 MB cells were derived from the malignant cells found in the pleural effusions of a 22-month old male patient and kindly provided by Dr. Matthias Wölfl. The D283 G3 MB cell line was established from malignant ascites cells and a peritoneal metastasis from a 6-year old male G3 MB patient and was purchased from the American Type Culture Collection (ATCC, Rockville, MD, USA). Human astrocytes were purchased from ScienCell Research Laboratories, Carlsbad, CA, USA. Human fetal neural stem cells were obtained from consenting patients, as approved by the Hamilton Health Sciences/McMaster Health Sciences Research Ethics Board and provided by Dr. Sheila Singh (McMaster University). A2780 (female) and HEYA8 (female) cells were provided by Dr. Mark Nachtigal (University of Manitoba). HCT116 (male) and SW480 (male) cells were provided by Dr. Kirk McManus (University of Manitoba) and MDA-MB-468 (female) cells were provided by Dr. Yvonne Myal (University of Manitoba).

Authentication

All cell lines have been authenticated by STR profiling (ATCC)

Mycoplasma contamination

All cell lines tested negative for Mycoplasma contamination using the MycoAlert® Mycoplasma Detection Kit (Lonza).

Commonly misidentified lines
(See ICLAC register)

No cell lines utilized in our study are listed in the ICLAC register

Animals and other research organisms

Policy information about studies involving animals; ARRIVE guidelines recommended for reporting animal research, and Sex and Gender in Research

Laboratory animals

Non-obese diabetic (NOD) severe combined immunodeficient (SCID) IL2R gamma null (NSG) mice (NOD.Cg-Prkdcscid Il2rgtm1Wjl/SzJ) purchased from Jackson Laboratories (Strain #:005557) aged 7-9 weeks were utilized for all in orthotopic xenograft in vivo analysis

Wild animals

Our study did not utilize wild animals

Reporting on sex

All medulloblastoma cell line models used in this study were of male origin and we utilized animals of matching sex (male) for all orthotopic xenograft in vivo studies. This is a disease that predominately afflicts male patients. However, main findings were validated in a variety of other cancer cell line including: colorectal cancer (HCT116 - male; SW480 - male), ovarian cancer (A2780 - female; HEYA8 - female) and breast cancer (MDA-MB-468 - female).

Field-collected samples

Our study did not involve field-collected samples

Ethics oversight

All experiments involving animals were approved by the University of Manitoba's Animal Care Committee (Protocol #21-021)

Note that full information on the approval of the study protocol must also be provided in the manuscript.

Flow Cytometry

Plots

Confirm that:

- [x] The axis labels state the marker and fluorochrome used (e.g. CD4-FITC).
- [x] The axis scales are clearly visible. Include numbers along axes only for bottom left plot of group (a 'group' is an analysis of identical markers).
- [x] All plots are contour plots with outliers or pseudocolor plots.
- [x] A numerical value for number of cells or percentage (with statistics) is provided.

Methodology

Sample preparation	To monitor apoptosis, cells were stained with the FITC Annexin V Apoptosis Detection Kit I from BD Biosciences according to manufacturers instructions. Live cells were suspended in 1X Annexin V binding buffer (0.1 M Hepes/NaOH (pH 7.4), 1.4 M NaCl, 25 mM $CaCl_2$) with 5µL of both Annexin.V and propidium iodide (PI) for 10 minutes and analyzed by flow cytometry using a BD FACS Canto-II.
Instrument	BD FACS Canto-II
Software	FlowJo™ v10 Software
Cell population abundance	Cell sorting was not performed in these studies, only analytical studies by flow cytometry
Gating strategy	For PI and Annexin V staining, "dead/dying" cells were not gated out, as they were included in the analysis. For PI incorporation, gates were set based on the 7AAD only negative control to determine the 'early apoptosis', 'late apoptosis', and 'necrosis' gate delineations. For Annexin V, gates were set based on 1. unstained controls. and 2. 7AAD only without Annexin V staining.

- [x] Tick this box to confirm that a figure exemplifying the gating strategy is provided in the Supplementary Information.

Magnetic resonance imaging

Experimental design

Design type	Resting state
Design specifications	N/A
Behavioral performance measures	N/A

Acquisition

Imaging type(s)	Structural
Field strength	7 Tesla
Sequence & imaging parameters	Fast spin echo T2, FOV 30 x 30 mm, matrix 256x 245, TR= 5000ms, TE=45ms, FA=90, coronal slice, 0.3mm slice thickness
Area of acquisition	Whole brain

Diffusion MRI [ ] Used [x] Not used

Preprocessing

Preprocessing software	N/A
Normalization	N/A
Normalization template	N/A
Noise and artifact removal	N/A
Volume censoring	N/A

Statistical modeling & inference

Model type and settings	N/A
Effect(s) tested	N/A

Specify type of analysis: ☒ Whole brain ☐ ROI-based ☐ Both

Statistic type for inference (See Eklund et al. 2016)	N/A
Correction	N/A

Models & analysis

n/a	Involved in the study
☒	☐ Functional and/or effective connectivity
☒	☐ Graph analysis
☒	☐ Multivariate modeling or predictive analysis
